# Cerebrospinal fluid and plasma concentrations of the inflammatory marker soluble CD27 in a large surgical population

**DOI:** 10.1016/j.isci.2024.110036

**Published:** 2024-05-21

**Authors:** Celien Tigchelaar, Janet L. Cunningham, Annica J. Rasmusson, Måns Thulin, Joachim Burman, Ido P. Kema, Anders Larsson, Anthony R. Absalom

**Affiliations:** 1 Department of Anesthesiology, University Medical Center Groningen, University of Groningen, Groningen, the Netherlands; 2 Department of Medical Sciences, Psychiatry, Uppsala University, Uppsala, Sweden; 3 Department of Mathematics, Uppsala University, Uppsala, Sweden; 4 Department of Medical Sciences, Neurology, Uppsala University, Uppsala, Sweden; 5 Department of Laboratory Medicine, University Medical Center Groningen, University of Groningen, Groningen, the Netherlands; 6 Department of Medical Sciences, Clinical Chemistry, Uppsala University, Uppsala, Sweden

**Keywords:** Health sciences, Medicine, Medical specialty, Internal medicine, Neurology, Neurosurgery

## Abstract

Soluble CD27 (sCD27) is a potential biomarker for diseases involving immune dysfunction. As there is currently little data on cerebrospinal fluid (CSF) sCD27 concentrations in the general population we measured CSF and plasma concentrations in 486 patients (age range 18–92 years, 57% male) undergoing spinal anesthesia for elective surgery. Across the complete cohort the median [range] sCD27 concentrations were 163 [<50 to 7474] pg/mL in CSF and 4624 [1830 to >400,000] pg/mL in plasma. Plasma sCD27, age and Qalb were the factors most strongly associated with CSF sCD27 levels. Reference sCD27 concentration intervals (central 95% of values) in a sub-group without the indication of neuropsychiatric, inflammatory or systemic disease (158 patients) were <50 pg/mL – 419 pg/mL for CSF and 2344–36422 pg/mL for plasma. These data provide preliminary reference ranges that could inform future studies of the validity of sCD27 as a biomarker for neuro- and systemic inflammatory disorders.

## Highlights

• CSF sCD27 correlates moderately with age and albumin quotient
• Plasma sCD27 is related to BMI, smoking, hematological and neuropsychiatric disorders
• Values were lower in a sub-cohort without systemic or neuropsychiatric disorders
• sCD27 upper reference estimates are 416 pg/mL in CSF and 36,422 pg/mL in plasma

## Graphical abstract

**Figure:**
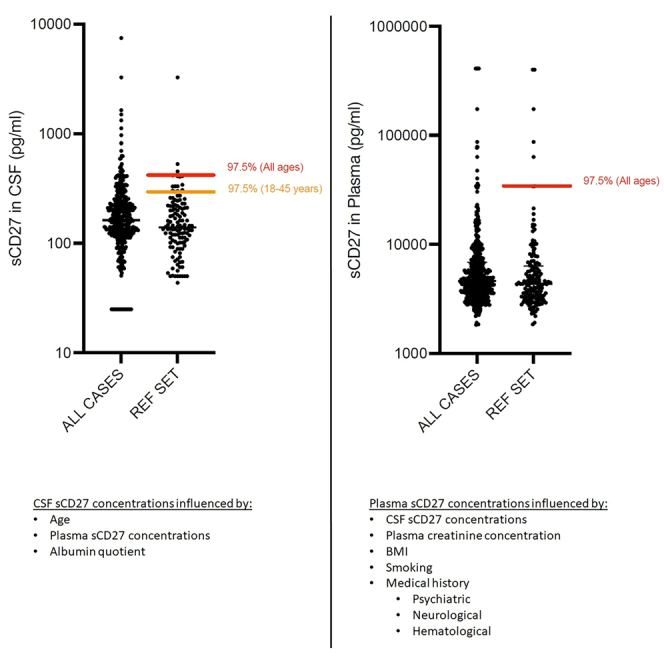

## Introduction

The soluble 32-kD form of CD27 (sCD27), which can be found in body fluids such as plasma and cerebrospinal fluid (CSF), has the potential to serve as a biomarker for diseases involving immune dysfunction and/or activation. CD27 belongs to the tumor necrosis factor (TNF) receptor family and is an antigen expressed primarily on naive peripheral blood T lymphocytes.^1^ When it binds CD70, its ligand on activated antigen-presenting cells, the T cell receptor is activated, expression on the cell membrane is upregulated and sCD27 is released.^1^^,^^2^ CD27 enhances T lymphocyte survival, expansion, differentiation and stimulates cytokine production.^1^ Therefore, sCD27 is regarded as a marker of adaptive immunity and has been studied primarily in diseases characterized by T cell activation.^3^ CD27 is also expressed on antigen-experienced B lymphocytes (memory B cells, which comprise 20–30% of B cells). Most evidence suggests that CD27 signaling promotes immunoglobulin production and B-cell differentiation,^1^^,^^4^^,^^5^ however, the function of the CD27^−^CD70 axis in B-lymphocytes is still poorly understood. To summarize, an inflammatory state can cause the up-regulation of CD27 expression and release of sCD27, inducing the activation and proliferation of T and B lymphocytes with the release of more pro-inflammatory cytokines.

Analysis of CSF sCD27 as candidate biomarker for central nervous system (CNS) disorders involving immune activation has mostly been performed in patients with neuroinflammatory disorders. In healthy states, entry of immune cells into the CNS is infrequent whereas lymphocytes and monocytes migrate across the blood-CSF-barrier during the course of CNS infections and inflammatory diseases such as multiple sclerosis (MS).^6^^,^^7^ Elevated sCD27 in CSF has been described and interpreted as a sign of intrathecal T cell–mediated inflammation in MS,^8^^,^^9^^,^^10^^,^^11^ Neuromyelitis Optica Spectrum Disorder^12^ and Huntington’s disease.^13^ A study by Komori et al. investigated multiple biomarkers of intrathecal inflammation and concluded that sCD27 was the best single biomarker.^9^

In addition to neuroinflammatory disorders, CSF sCD27 assays can help distinguish primary CNS lymphoma (PCNSL) from other types of brain tumors, which is very useful as this diagnosis can usually only be established with invasive histopathological techniques.^14^^,^^15^ CSF sCD27 has also been investigated as diagnostic tumor marker for the (lepto)meningeal involvement of lymphoid malignancies, but the evidence for this is contradictory.^14^^,^^16^^,^^17^ More in general, two studies have concluded that CSF sCD27 assays could discriminate between inflammatory and non-inflammatory neurological disorders, which can substantially assist the diagnosing process in this complex area of diseases.^8^^,^^18^

Plasma sCD27 has potential value as biomarker for immune activation and has been studied mostly in relationship with lymphoid malignancies and auto-immune disorders. Plasma sCD27 levels were elevated in patients with certain types of B-cell lymphomas,^19^^,^^20^^,^^21^ and correlated with tumor load, indicating that plasma sCD27 might be a disease-marker in patients with B-cell malignancies.^20^^,^^22^ As autoreactive lymphocytes are key factors in autoimmune disorders, elevated levels of serum sCD27 have been associated with thyroid disorders,^23^ systemic lupus erythematosus (SLE),^24^ psoriasis,^25^^,^^26^ inflammatory bowel disease (IBD)^27^ and rheumatoid arthritis.^28^ Moreover, sCD27 levels are elevated in patients with HIV^29^ and can even be used as marker for monitoring treatment response in HIV-1 infected patients.^3^ These prior studies demonstrate the high value of sCD27 as biomarker for immune activation.

The focus of most research so far has been to determine the value of sCD27 as biomarker for a specific disease. An essential step for identifying and validating sCD27 as biomarker in clinical practice is to explore the possible effect of biological factors such as age, comorbidity, blood-CSF-barrier (BCB) function and CSF inflammation on sCD27 concentrations.^30^^,^^31^ Only a few studies have investigated the correlation between biological factors and sCD27 levels, and then only as a secondary analysis.^8^^,^^11^^,^^12^^,^^14^ Even when the influence of factors, such as the IgG index, white cell count, and Interleukin 6 (IL-6) concentration have been studied, this has usually only been investigated in the disease group and not in the control group.^9^^,^^10^^,^^11^^,^^12^^,^^18^ Additionally, most studies performed so far included patients with neurological symptoms (e.g., lumbar disc protrusion, migraine or epilepsy) as control group due to the burden of a lumbar puncture (LP).^8^^,^^12^^,^^14^^,^^15^^,^^16^^,^^17^

The purpose of this study was to investigate plasma and CSF sCD27 concentrations in a relatively healthy surgical population, and to identify the correlations between physiological, social, cognitive and basic biochemical factors and sCD27 concentration. We also aimed to establish reference values for sCD27 concentrations in the whole cohort, which is representative of the general population, and in a control sub-cohort without the indication of neuropsychiatric, inflammatory or systemic disease.

## Results

### Study cohort and patient characteristics

Patient characteristics are shown in Table 1. The study cohort included 486 patients, 57% were male and the median age was 56 years (IQR: 38–68). Body mass index (BMI) ranged from 17 to 51 with a median of 27 kg/m^2^. The majority of patients were classified as American Society of Anesthesiologists (ASA) physical status I (43%) and II (44%), and the majority underwent orthopedic surgery (61%).

**Table 1:** Patient characteristics

N	486
**Demographics**	

Sex, male/female	277 (57%)/209 (43%)
Age (years)	56 [38–68]; range 18–92
Age categorical (years)	
18–19	15 (3%)
20–39	113 (23%)
40–59	153 (31%)
60–79	178 (37%)
80–99	27 (6%)
Ethnicity	
Caucasian	478 (98.4%)
African	4 (0.8%)
Asian	1 (0.2%)
Mixed	2 (0.4%)
Other	1 (0.2%)
BMI (kg/m^2^)	27 [24–31]; range 17–51

**ASA classification**	

ASA I	210 (43%)
ASA II	213 (44%)
ASA III	62 (13%)
ASA IV	1 (0.2%)

**Lifestyle factors**	

Smoking (*n* = 459)	
Never	281 (61%)
Current	76 (17%)
Former	102 (22%)
Alcohol use, yes/no (*n* = 444)	295 (66%)/149 (34%)
Drug use, yes/no (*n* = 451)	16 (4%)/435 (96%)
**Surgery** inpatient/outpatient	294 (60%)/192 (40%)

**Surgery type**	

Orthopedic	297 (61%)
Urological	100 (21%)
Surgical	55 (11%)
Gynecological	34 (7%)

Data are presented as mean ± SD, median [IQR] or frequency (%). BMI: Body Mass Index. ASA: American Society of Anaesthesiologists.

A summary of relevant patient medical history, medication use and neurocognitive function is provided in Table 2. MOCA scores were available in 250 patients. The median MoCA test score was 26 (IQR: 24–28), and 59% of patients had a normal MoCA score (≥26). Of the 486 patients, 78% used medication and 94% had at least one (past) co-morbid condition (other than the indication for surgery). Most frequently, patients had a disorder of the locomotor system (59%). A spinal disc herniation was the most common neurological problem and depression the most common psychiatric problem. Of the total group, 51 patients (10%) had at least one autoimmune disorder. Four patients were pregnant (between 13 and 15 weeks gestation) at the time of biomaterial collection (median age: 31 years). Aspects of the neurological examination (as assessed by medical students) were abnormal for 17%–65% of the patients, although in no cases did the findings suggest a neurological disorder requiring a new referral or further investigation. The detailed results for all 26-items of the neurological examination can be found in Table S1.

**Table 2:** Medical history, medication use and neurological and cognitive function

N	486
**Medical history**	455 (94%)
General	267 (55%)
*(Seropositive) Rheumatoid Arthritis*	17 (3%)
*Psoriasis vulgaris*	10 (2%)
*Dermatopolymyositis*	3 (0.6%)
*Ankylosing Spondylitis (AS)*	3 (0.6%)
*Iridocyclitis*	2 (0.4%)
*Pernicious Anemia*	1 (0.2%)
*Pemphigoid*	1 (0.2%)
*Wegener’s Granulomatosis*	1 (0.2%)
*Polymyalgia Rheumatica*	1 (0.2%)
*Myasthenia Gravis*	1 (0.2%)
*Sjogren’s Syndrome*	1 (0.2%)
Cardiovascular	187 (38%)
Respiratory	102 (21%)
Gastrointestinal	113 (23%)
*Inflammatory bowel disorder (IBD)*	7 (1%)
*Primary Biliary Cirrhosis*	1 (0.2%)
Neurological	93 (19%)
Spinal disc herniation	25 (5%)
CVA/TIA	18 (4%)
Migraine	8 (2%)
Meningitis	5 (1%)
Parkinson disease	5 (1%)
Epilepsy/seizures	4 (0.8%)
Pituitary adenoma	4 (0.8%)
Schwannoma	4 (0.8%)
Multiple Sclerosis	1 (0.2%)
Dementia/Alzheimer disease	1 (0.2%)
Endocrinological	106 (22%)
*Thyrotoxicosis*	3 (0.6%)
*Type 1 Diabetes*	2 (0.4%)
*Autoimmune Thyroiditis*	1 (0.2%)
Urological	110 (23%)
Locomotor	286 (59%)
Hematological	18 (4%)
Psychiatric	44 (9%)
Depression	26 (5%)
Anxiety disorder	11 (2%)
Alcohol/drug abuse	8 (2%)
ADHD	5 (1%)
Suicide attempt/suicidal behavior	2 (0.4%)
Psychosis/Schizophrenia	2 (0.4%)
Personality disorder	2 (0.4%)
**Medication use**	379 (78%)
**MoCA score** (*n* = 250)	26 [24–28]; range 14–30
**Neurological exam** (*n* = 242)	Abnormal findings:
Cranial nerve function	65 (27%)
Motor function	74 (31%)
Sensory function	106 (44%)
Coordination	41 (17%)
Gait	77 (32%)
Reflexes	158 (65%)

Data are presented as median [IQR] or frequency (%). For medical history, the number of patients are shown, and autoimmune diseases are shown in italics. CVA: Cerebro Vascular Accident; TIA: Transient Ischemic Attack; MDD: Major Depressive Disorder; ADHD: Attention Deficit Hyperactivity Disorder; MoCA: Montreal Cognitive Assessment.

### Cerebrospinal fluid and blood collection

Of the total of 486 patients, the planned spinal anesthetic procedure was not completed for various reasons in twelve patients. In sixteen patients the spinal puncture failed completely. In twelve patients the intrathecal space was identified but CSF aspiration failed. In the remaining 446 patients, a mean of 9.3 mL CSF (range 0.0–13.0) was collected over an average of 2 min. As the volume of CSF collected was less than 10 mL in some cases, CSF was available for *routine* analysis for 440/441 patients and for *sCD27* analysis for 405 patients. Blood was collected in 481 patients, of whom blood from 480 was available for storage and 478 for routine analysis.

### Laboratory analysis: plasma and cerebrospinal fluid soluble CD27 concentrations

Results of leukocyte count, erythrocyte count (only CSF), albumin, total protein, glucose, sCD27, CRP (plasma) and creatinine (plasma) concentrations are shown in Table 3. CSF leukocyte count ranged from 0 to 59 x10^6^/L with a median of 1.0. The median CSF erythrocyte count was 200/μL and in 80 patients (18%) this count exceeded 500/μL. The median Qalb value was 5.5 x10^−3^ (IQR: 3.8–7.6). Median plasma CRP was 1.26 mg/L and in 19% of cases CRP was below the LOD.

**Table 3:** Plasma and CSF laboratory analysis

	n	Median [IQR]	Range
**Plasma**			

Leukocyte count (x10^9^/L)	473	6.4 [5.4–7.8]	2.9–35.7
Albumin (g/L)	478	44 [42–46]	20–56
Total protein (g/L)	478	72 [69–75]	33–97
Glucose (mmol/L)	473	5.4 [5.0–5.9]	3.7–17.2
CRP (mg/L)	478	1.26 [0.31–3.39]	<0.2 (0.1) – 113.3
Creatinine (umol/L)	478	76 [65–89]	34–565

**CSF**			

Leukocyte count (x10^6^/L)	440	1.0 [1.0–2.0]	0–59
Erythrocyte count (x10^6^/L)	440	200 [100–400]	0–32000
Albumin (g/L)	440	0.24 [0.18–0.33]	0.06–1.00
Total protein (g/L)	441	0.38 [0.30–0.50]	0.14–1.45
Glucose (mmol/L)	441	3.4 [3.2–3.6]	1.1–9.1
Qalb (x10^−3^)	433	5.5 [3.8–7.6]	1.4–21.7

Results of plasma and CSF sCD27 assays are shown in Table 4. For the total cohort, the median [range] CSF sCD27 concentration was 163 [<50–7474] pg/mL and the median [range] plasma sCD27 concentration was 4624 [1830 - >400,000] pg/mL. In the total cohort CSF sCD27 concentrations were below the LOD of 50 pg/mL in 5%, and plasma sCD27 levels exceeded the upper limit of quantification of 400,000 pg/mL in 1% of the patients.

**Table 4:** Results of plasma and CSF sCD27 assays and derived results

	Total cohort			Control sub-population		
	n	Median [IQR]	Reference interval	n	Median [IQR]	Reference interval
**Plasma**						

sCD27 (pg/mL)						
All ages	478	4624 [3570–6860]	2467–40,791	158	4322 [3280–6080]	2344–36,422
18–44 years	143 (30%)	4472 [3364–4467]	2327–78.298	72 (46%)	4537 [3025–6889]	2340–68,544
45–64 years	179 (37%)	4478 [3541–6117]	2493–34,423	46 (37%)	3991 [3321–5765]	2718–24,538
65–92 years	156 (33%)	5019 [3797–6972]	2553–23.686	19 (15%)	4293 [3695–5843]	2199–26,532

**CSF**						

sCD27 (pg/mL)						
All ages	405	163 [120–231]	<50–625	125	139 [101–218]	<50–419
18–44 years	124 (31%)	136 [112–207]	<50–373	60 (48%)	124 [91–179]	<50–289
45–64 years	150 (37%)	162 [118–226]	<50–624	46 (37%)	144 [112–199]	<50–406
65–92 years	131 (32%)	201 [134–317]	<50–786	19 (15%)	272 [146–373]	<50 - 437

**Normalized CSF sCD27 values**						

QsCD27 (x10^−3^)						
All ages	398	32.8 [18.7–50.9]	4.1–124.7	123	33.5 [17.3–50.2]	5.4–98.5
18–44 years	119 (30%)	28.7 [14.8–42.0]	4.1–72.3	58 (47%)	26.9 [15.0–39.8]	4.9–29.7
45–64 years	148 (37%)	34.4 [18.6–51.6]	3.9–125.9	46 (37%)	39.4 [20.3–50.6]	5.5–135.7
65–92 years	133 (33%)	34.6 [22.8–59.7]	5.1–128.8	19 (15%)	45.3 [24.5–73.7]	9.1–95.2
QsCD27/Qalb						
All ages	394	5.6 [3.2–9.1]	0.7–26.7	118	5.9 [3.2–10.5]	0.7–25.8
18–44 years	117 (30%)	6.2 [3.7–9.5]	0.9–20.0	57 (48%)	5.6 [3.2–8.3]	0.8–19.9
45–64 years	143 (37%)	5.6 [3.1–10.4]	0.4–25.7	42 (36%)	5.7 [2.6–11.9]	0.3–33.4
65–92 years	127 (33%)	5.3 [3.0–8.5]	0.7–32.9	19 (16%)	7.5 [5.0–11.7]	0.9–17.6

CSF sCD27 LOD 50 pg/mL (< LOD replaced with LOD/2; 5% of data); Plasma sCD27 DL 400,000 (DL replaced with 401,000; 1% of data); Plasma CRP LOD 0.2 mg/L (< LOD replaced with LOD/2 = 0.1; 19% of data). CSF: Cerebrospinal Fluid. Qalb: CSF albumin/plasma albumin. QsCD27: CSF sCD27/plasma sCD27.

The control subpopulation without known comorbidity comprised 158 patients for whom CSF was available in 125 and plasma in 158 patients. In this control subpopulation the median [range] CSF sCD27 concentration was 139 [<50–3267] pg/mL and median [range] plasma sCD27 4322 [1851 - >400,000] pg/mL.

Results of CSF sCD27 assays normalized for plasma sCD27 (QsCD27), and normalized for plasma sCD27 and Qalb (QsCD27/Qalb), for the total cohort and the control sub-population, are shown in Table 4. QsCD27/Qalb values ranged from 0.06 to 56.7.

### Factors related to soluble CD27 concentrations across the total cohort

**Figure 1:**
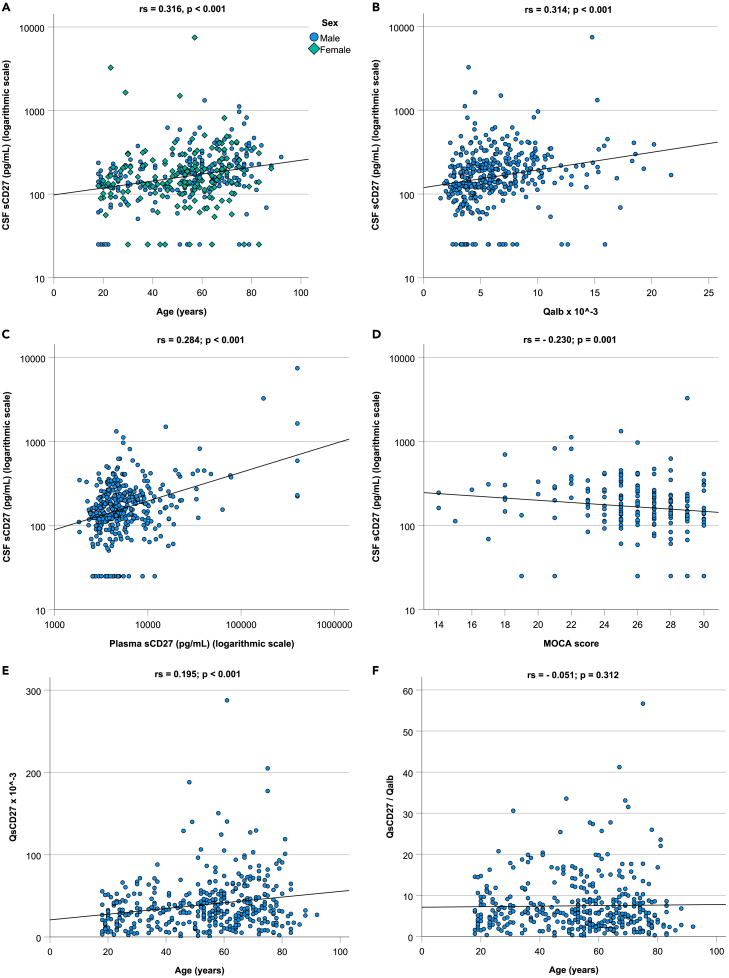
Scatterplots showing correlations between CSF sCD27 concentrations and various factors Correlations between CSF sCD27 and (A) age (*n* = 405), (B) Qalb (*n* = 397), (C) plasma sCD27 (*n* = 397), and (D) MoCA score (*n* = 206); and (E) between QsCD27 and age (*n* = 397) and (F) between QsCD27/Qalb ratio and age (*n* = 394). Co-efficients and *p* values calculated using (Lasso (least absolute shrinkage and selection operator) regression analysis). Qalb: CSF albumin/plasma albumin. QsCD27: CSF sCD27/plasma sCD27. MoCA: Montreal Cognitive Assessment.

The associations between CSF sCD27 concentrations and physiological, social, cognitive pre-analytical and basic biochemical factors in the total study group are shown in Table 5 and Figure 1. There was a moderate positive correlation between CSF sCD27 and age (r_s_ = 0.316, *p* < 0.001) and between CSF sCD27 and Qalb (r_s_ = 0.314: *p* < 0.001). Also, BMI (r_s_ = 0.142, *p* = 0.004), plasma leukocyte count (r_s_ = 0.107, *p* = 0.034), CSF leukocyte count (r_s_ = 0.142, *p* = 0.004), CSF albumin (r_s_ = 0.287, *p* < 0.001), plasma sCD27 (r_s_ = 0.284, *p* < 0.001), plasma CRP (r_s_ = 0.168, *p* = 0.001) and plasma creatinine (r_s_ = 0.135, *p* = 0.007) were significantly correlated with CSF sCD27. Preoperative MoCA scores were inversely correlated with CSF sCD27 (r_s_ = −0.230: *p* = 0.001; *n* = 206).

**Table 5:** The association between CSF sCD27 concentrations and physiological, social, cognitive pre-analytical and basic biochemical factors

Study group (*n* = 405)	Median [IQR] or frequency (%)	CSF sCD27 median [IQR] (pg/mL)	Correlation with CSF sCD27	
			r_s_	*p* value
Age (years)	56 [37–68]		0.316	<0.001∗∗∗
Sex				
Male	233 (58%)	171 [122–244]		0.116
Female	172(42%)	148 [111–217]		
BMI (kg/m^2^)	27 [24–30]		0.142	0.004∗∗
Alcohol use (*n* = 370)				
Yes	242 (65%)	161 [120–236]		0.888
No	128 (35%)	169 [112–230]		
Smoking (*n* = 380)				
Yes	59 (16%)	166 [118–231]		0.887
No	321 (84%)	161 [120–237]		
Plasma haemolysis (*n* = 397)				
Yes	50 (13%)	153 [117–234]		0.621
No	347 (87%)	161 [120–232]		
CSF erythrocytes				
0–500	335 (83%)	161 [121–237]		0.329
>500	70 (17%)	163 [102–220]		
Medical history				
Yes	376 (93%)	165 [120–240]		0.018∗
No	29 (7%)	134 [91–190]		
General				
Yes	224 (55%)	185 [123–254]		0.004∗
No	181 (45%)	147 [112–208]		
Cardiovascular				
Yes	150 (37%)	194 [137–266]		<0.001∗∗∗
No	255 (63%)	144 [112–219]		
Respiratory				
Yes	86 (21%)	171 [121–228]		0.651
No	319 (79%)	161 [120–237]		
Gastrointestinal				
Yes	91 (23%)	182 [123–244]		0.468
No	314 (77%)	161 [120–230]		
Neurological				
Yes	80 (20%)	182 [123–254]		0.098
No	325 (80%)	161 [120–227]		
Endocrinological				
Yes	88 (22%)	195 [135–293]		0.002∗∗
No	317 (78%)	157 [115–225]		
Urological				
Yes	96 (24%)	194 [136–273]		0.005∗
No	309 (76%)	154 [118–223]		
Locomotor				
Yes	229 (56%)	163 [122–246]		0.405
No	176 (44%)	157 [112–231]		
Hematological				
Yes	17 (4%)	199 [135–266]		0.309
No	388 (96%)	161 [120–231]		
Psychiatric				
Yes	35 (9%)	183 [121–263]		0.298
No	370 (91%)	161 [120–231]		
Autoimmune diseases				
Yes	44 (11%)	203 [135–310]		0.008∗∗
No	361 (89%)	161 [118–228]		
Medication use				
Yes	320 (79%)	179 [123–254]		<0.001∗∗∗
No	85 (21%)	134 [108–182]		

Test is significant at 0.05 level (∗),0.01 level (∗∗) or 0.001 level (∗∗∗) (two-tailed). r_s_: Spearman correlation coefficient. BMI: Body Mass Index. Qalb: CSF albumin/plasma albumin. MoCA: Montreal Cognitive Assessment.

There was no significant difference in CSF sCD27 concentrations between patients with a CSF erythrocyte count below 500 x10^6^/L and exceeding 500 x10^6^/L (*p* = 0.329), and CSF erythrocyte count was not significantly correlated with CSF sCD27 (r_s_< 0.001: *p* = 0.993). Multiple medical history categories were significantly related with CSF sCD27, including having at least one general (*p* = 0.004), cardiovascular (*p* < 0.001), endocrinological (*p* = 0.002) or urological (*p* = 0.005) co-morbidity. Patients using no medication at all had a significantly lower CSF sCD27 than patient using at least one medication (*p* < 0.001). Smoking and alcohol consumption did not have a significant association with sCD27. The CSF sCD27 concentration of pregnant patients (median: 212 pg/mL) (*n* = 3) was not significantly different compared to age-matched (20–39 years old) nonpregnant women (median: 134 pg/mL, *n* = 39) (*p* = 0.807).

The associations between plasma sCD27 concentrations and physiological, social, cognitive, pre-analytical and basic biochemical factors are shown in Table S2. Age (*p* = 0.014), plasma albumin (*p* = 0.002), plasma CRP (*p* = 0.037) and plasma creatinine (*p* < 0.001) were significantly associated with plasma sCD27. Also, an endocrinological (*p* = 0.043), urological (*p* = 0.022) and hematological (*p* = 0.003) medical history were related to plasma sCD27. The plasma sCD27 concentration of pregnant patients (median: 3327 pg/mL) (*n* = 3) did not differ with age-matched nonpregnant women (4567 pg/mL) (*n* = 49) (*p* = 0.263).

### Predicting soluble CD27 concentrations with a multivariable model

Of the twenty-six explanatory variables that entered the Bayesian lasso regression model for CSF sCD27, twenty-three variables were removed. The three remaining predicting variables in the model were (from most important to least important): plasma sCD27, age and Qalb (*n* = 389 patients, mean of log 2 CSF sCD27 = 7.34, standard deviation (SD) = 1.07, the average prediction error (RMSE) = 1.0092664). Plasma sCD27 (P(β≠0|x) = 1.000), age (P(β≠0|x) = 0.975) and Qalb (P(β≠0|x) = 0.928) had the highest posterior effects (see Table S3; Figure S1).

For plasma sCD27, seven out of twenty-six variables remained in the final model (*n* = 389, mean of log2 plasma sCD27 = 12.45, SD = 1.12, RMSE = 1.0703989). These variables include (in order of importance): CSF sCD27, plasma creatinine, BMI, smoking, a psychiatric medical history, neurological medical history and hematological medical history. The results of the Bayesian regression models can be found in Table S4 and Figure S2.

### Reference intervals for soluble CD27

Reference intervals for the total cohort and control subpopulation are shown in Table 4. In the total cohort, the 2.5^th^ – 97.5^th^ percentiles of CSF sCD27 were < LOD (50 pg/mL) – 625 pg/mL (*n* = 405) and of plasma sCD27 were 2,467–40,791 pg/mL (*n* = 478). Of the total study group, 158 patients fulfilled the criteria for being healthy (median [IQR] age: 49 [31–58] years. In the control subpopulation the 2.5^th^ – 97.5^th^ percentiles of CSF sCD27 were < LOD (50 pg/mL) – 419 pg/mL (*n* = 125) and of plasma sCD27 were 2,344–36,422 pg/mL (*n* = 158).

## Discussion

sCD27 has been proposed as a potential biomarker of neuroinflammation. To better understand the diagnostic and prognostic value of plasma and CSF sCD27 concentrations, as with all biomarkers, knowledge of the range and variability in these concentrations among the general population is needed. We have therefore measured concentrations of the candidate biomarker sCD27 in CSF and plasma of a population considered fit for elective surgery under spinal anesthesia. We then determined and report here age-related preliminary reference values for the total cohort (a relatively healthy surgical cohort of 486 patients with an age range from 18 to 92 years old, the majority of whom underwent orthopedic surgery) and also for a control sub-population (comprising 158 patients with an age range from 18 to 87 years, and no evidence of any systemic or neurological diseases). Further analyses were performed to determine sources of variability in sCD27 concentrations in the complete cohort. In particular we examined correlations among physiological and biochemical factors influencing sCD27 concentration. A multivariable model showed that the most important factors explaining variability in CSF sCD27 concentrations were plasma sCD27, age and Qalb. For plasma sCD27 concentrations, predictive factors included CSF sCD27, plasma creatinine, BMI, smoking, and a psychiatric-, neurological- and hematological medical history.

As has been mentioned above, CSF sCD27 concentration analyses have previously mostly been performed in patients with neuroinflammatory disorders without an adequate reference group. Most previous studies aimed to identify or validate CSF sCD27 as marker to distinguish between inflammatory or other neurological diseases and healthy condition,^8^^,^^9^^,^^10^^,^^12^^,^^14^^,^^15^^,^^18^ or to investigate the predictive value of sCD27 for the course of a specific disease such as MS.^11^^,^^13^ Frequently, these studies have not clearly stated concentrations of CSF sCD27 for the control group.^9^^,^^10^^,^^14^^,^^16^^,^^17^ Therefore, healthy control reference concentrations of CSF sCD27 are scarce or difficult to compare between studies, particularly as most previous studies with control data expressed concentrations of CSF sCD27 as units per milliliter (U/mL), which makes the result highly dependent on the specific assay used.^8^^,^^11^^,^^12^^,^^14^^,^^15^

There are a few exceptions however. Feresiadou et al. reported the results of CSF sCD27 concentration assays in one of the largest cohorts published so far, comprising 676 patients with neurological disorders and 127 controls.^18^ The control group included healthy volunteers (*n* = 47), symptomatic patients in whom a neurological disorder was ruled out (*n* = 38) and patients who underwent spinal anesthesia for urological surgery (*n* = 42). Among all the control subjects, the median CSF sCD27 concentration was 64 pg/mL; in the healthy volunteers it was 16 pg/mL, and among patients in whom a neurological disorder was ruled out it was 13 pg/mL, whereas in urological controls it was 252 pg/mL. The majority of patients in our cohort were labeled as having ASA physical status I or II, meaning they are either “fit and healthy patients” or “patients with mild systemic disease,” nonetheless across the whole cohort, and even among the selected control subpopulation the median sCD27 concentrations (163 and 143 pg/mL respectively) were far higher than among the non-surgical controls, but somewhat lower than the concentrations of the urological patients, in the study of Feresiadou (252 pg/mL) who had multiple co-morbidities. Even though our patients were considered fit for surgery, as they all had an indication for surgery, it is likely that their surgical problem, combined with the comorbidities among those who were less fit, were the cause of a higher sCD27 concentration than among the healthy volunteers of Feresiadou. On the other hand, the mean values in our cohort were lower than those found among 35 controls in a recent study of MS biomarkers, but it is important to note that although these “control” patients did not have MS, they were symptomatic (sCD27 concentrations were ±300 pg/mL; actual values only shown in a figure).^10^ It is reassuring too that the upper limit of CSF sCD27 concentrations found in our healthy subpopulation (516 pg/mL) was lower than the median sCD27 concentration of 740 pg/mL found among patients with neurological disorders with an inflammatory component in the study of Feresiadou et al.^18^

In our cohort age, Qalb and plasma sCD27 concentration were the most important factors associated with CSF sCD27 concentrations. These findings were surprising, as they are contrary to what has been reported in literature. One explanation for this inconsistency could be that the factors influencing sCD27 concentrations have mostly been studied in patients with neuroinflammatory disorders and not healthy controls.^8^^,^^10^^,^^11^^,^^12^ Secondly, most studies did not primarily investigate the influence of biological factors, and the small sample sizes may have been too low to provide power for analyses.^8^^,^^11^^,^^12^^,^^14^^,^^15^^,^^16^ For age, three previous studies have found no correlation, in both diseased patients and in healthy controls.^9^^,^^10^^,^^18^ This contrary finding could be attributed to the fact that the correlation we have found were weak to moderate and the age distributions of study populations varied considerably. Our study included a large group with ages ranging from 18 to 92 years old but previous studies usually only included a small group of patients with a limited age range (usually between 30 and 60 years old). Additionally, our sample is probably healthier compared to prior studies since these studies included mostly symptomatic and diseased patients.^9^^,^^10^

Aging usually is associated with systemic chronic inflammation, so-called “inflammaging,” which among other effects increases the percentage of B cells in plasma expressing CD27.^20^^,^^32^^,^^33^ There is always a gradient between plasma and CSF sCD27 concentrations with much higher concentrations in the plasma, and we have shown that CSF sCD27 levels correlate weakly with plasma sCD27 levels. This contrasts with the findings of two previous studies that showed no correlation.^8^^,^^14^ This discrepancy between could be attributed to the larger population, fact that both previous studies included unhealthy patients as well as symptomatic controls with other neurological diseases, and they did not take into account the effect of BCB permeability.

“Inflammaging” is likely to not only increase plasma sCD27 concentrations but also increase BCB permeability,^34^ with both factors contributing to an increase in CSF sCD27 concentrations. Whereas two previously conducted studies found no correlation between Qalb (a marker of BCB permeability) and CSF sCD27 concentrations,^8^^,^^9^ we have found a moderate correlation between CSF sCD27 and Qalb (Figure 1B; Table 5). When CSF sCD27 values were normalized for plasma sCD27 concentrations the correlation between CSF sC27 and age persisted, but when CSF sCD27 concentrations were normalized to plasma sCD27 concentrations and Qalb, the correlation with age was no longer present, most likely because Qalb increases with age.

Inflammatory markers such as leukocyte and CRP concentrations are expected to be associated with sCD27 concentrations. Our results showed very weak univariate correlations between CSF sCD27 and CRP, plasma and CSF leukocyte concentrations. These effects were not significant in the multivariable model, possibly due to the low number of patients in our cohort with an infection and/or CSF pleocytosis. All previous studies investigated only univariate correlations.^8^^,^^9^^,^^10^^,^^11^^,^^12^^,^^18^ In accordance with the present results, a previous study has demonstrated that CSF sCD27 concentrations correlate with absolute numbers of T cells.^9^ For WBC count, results were contradictory.^9^^,^^10^^,^^12^ To the best of our knowledge, no evidence is available regarding the relationship between CSF sCD27 and CRP. One study using serum found a correlation between **serum** sCD27 and CRP.^35^

Compared to CSF sCD27, more factors were significantly correlated with plasma sCD27 levels. This finding could indicate that CSF sCD27 is less dependent on biological variation compared to plasma sCD27. Evidence regarding the relationship between biological factors and plasma sCD27 is very limited. Our study found similar results to those of previous studies regarding the relationship between plasma sCD27 and sex^24^ and CRP.^27^ On the other hand, our study results were contrary to what has been reported for the association between plasma sCD27 and age,^36^ BMI,^36^ plasma leukocytes,^27^ smoking^36^ and CRP.^35^ Discrepancies between our study and previous studies could partly be explained by the fact that most previous studies included diseased patients and no healthy controls, sample sizes were smaller and all studies performed univariate analyses instead of building a multivariable model.

The large majority of patients in our complete cohort showed no reason to suspect neuronal inflammation other than mild inflammation associated with aging, and so variations in CSF sCD27 were therefore likely largely caused by age-related BCB permeability changes, and by differences in plasma sCD27 concentrations influenced by systemic and biological factors. Our data therefore suggest that in neurologically unwell patients, a raised CSF sCD27 in the presence of a normal plasma sCD27 will be highly suggestive of a neuroinflammatory disorder. In patients with known or suspected neuroinflammatory disorders, plasma sCD27 concentrations may also be elevated; but when CSF sCD27 concentrations are normalized or corrected for plasma sCD27 and Qalb, and still remain above the reference ranges, this too will be highly suggestive of a neuroinflammatory disorder. In particular, the combined measurement of Qalb, CSF and plasma sCD27 concentrations have been postulated to have clinical utility in the diagnosis and disease monitoring activity of Multiple Sclerosis,^8^^,^^9^^,^^10^^,^^11^^,^^37^ and Neuromyelitis Optica syndromes^12^ and in primary CNS lymphomas^15^ although further research is needed, using standardized assays, concentration units and reporting standards, along with analysis of sensitivity and specificity. There may plausibly be a potential role of combined CSF and plasma sCD27 assays in the diagnosis and monitoring of cases of encephalitis (whether viral – such as HIV – or auto-immune in origin, such as in anti-NMDA receptor encephalitis), Alzheimer’s Disease and Parkinson’s Disease. When clinicians interpret patient test results by comparing them with those of a reference or control population, it is important that the reference population is relevant to their specific patient. In our study, our complete cohort comprised a surgical population of patients with a wide range of ages who were undergoing elective surgery under spinal anesthesia. Although this group comprised patients with a wide range of ages, and a wide range of health conditions, as mentioned above, 87% of patients in the complete cohort, and all of the patients in the control sub-cohort, were considered by the responsible anesthesiology team to have ASA status I or II (patients with status I are “fit and healthy” and patients with status II have “mild systemic disease” without “substantial functional limitations”). We believe that the complete cohort is relevant to the general population, and that the control sub-cohort is an appropriate choice of control population for CSF sCD27 analyses, as it comprises individuals with no indication of neuropsychiatric, inflammatory or systemic disease, but is still representative of a real life population, with more occasional minor health conditions (such as asthma) not interfering with their normal activities.

This study has several strengths. The first strength is the wealth of metadata we collected, among which were the results of a neurological examination, the results of MOCA scores, and granular details on past medical and medication history. Secondly, our sample is one of the largest in this research area and represents a broad selection from a surgical population which is probably reasonably representative of the general population. This is supported for instance by the fact that the incidence of comorbidities in this study corresponds broadly with the incidence of diseases in the general population,^38^ cognitive scores were comparable with those in the general population^39^ and CSF composition was normal (routine CSF analyses were in the normal range). Third, due to the large sample size and broad variation in factors, for example for age (18–92 years), BMI (17–51 kg/m^2^) and Qalb (1.4–21.7 x10^−3^), we had sufficient power to not only perform univariate analyses but also add multivariate analyses so we were able to detect the effect of the most important biological factors on sCD27 concentrations.^30^^,^^31^ Also, this is one of the few studies examining both CSF and plasma, and interesting differences have been found. Lastly, biomaterial was obtained using highly standardized collection methods^40^ and all analyses were performed within the same batch. So far, single CSF biomarkers have limited clinical value and the past decades few new markers have been identified.

In conclusion, this study demonstrated that several biological factors such as age, BMI, Qalb and certain comorbidities are associated with sCD27 concentrations in CSF and plasma. This exploratory study is an important first step for identifying and validating sCD27 as biomarker for neuroinflammatory disorders. Future studies should take the biological variation of sCD27 concentrations into account and focus on studying the relevance of sCD27 as biomarker in a clinical setting, by investigating if sCD27 is superior to current biomarkers and diagnostic procedures for the identification of neuroinflammation.

### Limitations of the study

Our sample is selective and consists of patients undergoing spinal anesthesia. As they had an indication for surgery, they could be considered to be a not completely healthy control group, even though the majority were undergoing orthopedic procedures for problems not caused by ill-health.

We have found multiple factors influencing sCD27 concentrations such as age, but were restricted due to our sample size to correct our reference ranges for these patient characteristics (e.g., multiple reference sample groups stratified by age).^30^ This may be remedied in the future by using a larger sample size.

IgG index and other possible important inflammatory markers were not studied due to the setup of the original study.

Although all comorbidities of the patients were registered, these conditions were categorized by one of the two coordinating investigators and decisions could vary slightly between the two, so results of this part of our study therefore need to be interpreted with caution. Also, the numbers of patients with an abnormal neurological exam recorded by medical students were relatively high, but this is (partly) due to our scoring system which may have been over-inclusive: if one of the items of that aspect (e.g., reflexes) was abnormal, then the complete category was marked as "abnormal”.

## STAR★Methods

### Key resources table

**Table:** 

REAGENT or RESOURCE	SOURCE	IDENTIFIER
**Biological samples**		

Human patient plasma samples	Anesthetic Biobank of Cerebro-spinal Fluid, University Medical Center Groningen, The Netherlands	Cohort and Biobanking Coordination Hub (CBCH), UMCG Groningen, reference 201600271
Human patient cerebrospinal fluid samples	Anesthetic Biobank of Cerebro-spinal Fluid, University Medical Center Groningen, The Netherlands	Cohort and Biobanking Coordination Hub (CBCH), UMCG Groningen, reference 201600271

**Critical commercial assays**		

sCD27 assays	Sandwich ELISA kit (DY382, R&D Systems, Minneapolis, MN, USA)	Cat# DY382
Plasma creatinine assays	IDMS calibrated enzymatic creatinine reagents from Abbott Laboratories (Abbott Park, IL, USA); analysis on a BS380 analyser (Mindray, Shenzhen, China).	Cat# 8L24-41
High-sensitivity CPR assays	Plasma hs-CRP was analysed with reagents from Abbott Laboratories (Abbott Park, IL, USA) on a BS380 analyser (Mindray, Shenzhen, China)	Cat# 6K26-41

**Deposited data**		

Clinical patient data	Anesthetic Biobank of Cerebro-spinal Fluid, University Medical Center Groningen, The Netherlands	Cohort and Biobanking Coordination Hub (CBCH), UMCG Groningen, reference 201600271

**Software and algorithms**		

SPSS statistics software (version 23.0)	IBM Corp., Armonk, New York, Unites Stated of America	https://www.ibm.com/spss
R Core Team (2018)	R Foundation for Statistical Computing, Vienna, Austria.	https://www.R-project.org/

### Resource availability

#### Lead contact

Further information and requests for resources and reagents should be directed to and will be fulfilled by the lead contact, Anthony Absalom (a.r.absalom@umcg.nl).

#### Materials availability

This study did not generate new unique reagents.

#### Data and code availability

All data reported in this paper – the clinical data held in the Anaesthetic Biobank of Cerebrospinal Fluid, and the results of laboratory assays - will be shared by the lead contact upon request.

No original code has been generated in this work.

Any additional information required to re-analyze the data reported in this paper is available from the lead contact upon request.

### Experimental model and study participant details

#### Study design and ethics

This study used biomaterial and data from patients enrolled in the Anaesthetic Biobank of Cerebrospinal Fluid (ABC) study of the University Medical Center Groningen (UMCG), The Netherlands, between October 2016 and November 2020. CSF and blood were collected from patients undergoing planned spinal anesthesia for elective surgery. The purpose of the ABC was to collect and store CSF from patients without major neurological disease for future neuroscientific research. The ABC was approved by the Medical Ethical Committee of the UMCG (registration number 2016–174), conforms to the provisions of the Helsinki Declaration as revised in 2013; written informed consent was obtained from all participants.

#### Overview of main study population, material collection and measurements

More extensive details of the study design, patient inclusion and data and biomaterial collection are described elsewhere.^40^ All patients aged 18 years and older scheduled for elective surgery under spinal anesthesia (except for caesarean section) were invited to participate in the biobank. Patients were screened for eligibility to undergo spinal anesthesia. The absolute contraindications for spinal anesthesia were an increased intracranial pressure and local skin infection (risk of meningitis). Relative contraindications included a neurological deficit or demyelinating disease, uncorrected hypovolemia, sepsis, coagulopathy or use of anticoagulants, fixed cardiac output states (e.g. aortic stenosis) and spinal deformities. Also, contraindications for elective surgery are clinical signs of infection such as fever, cough or tachycardia.

Data from all patients who were enrolled in the study, and in whom CSF and/or plasma was acquired, was used for the current study. In total the data from 486 patients have been analysed for the current study, among whom the median [IQR](range) age was 56 [38 – 68(18 – 92); and among whom 277 (57%) were male and 209 (43%) were female.

### Method details

#### Tissue samples and clinicopathological data

##### CSF and blood collection and basic analyses

The spinal puncture and collection of biomaterials were carried out according to standardized procedures.^40^ Ten mL of CSF was aspirated into five consecutive two mL syringes during the spinal puncture prior to local anaesthetic injection. The first two mL of CSF was immediately used for routine analyses, including albumin, total protein, glucose, leukocyte and erythrocyte counts. The remaining CSF was centrifuged (1000 x g, 10 minutes, 4ºC) and stored at -80ºC in aliquots of 500 μL. Prior to surgery, during intravenous cannulation, 20.5 mL of blood was collected. Part of the blood was directly used for routine analyses (albumin, total protein, glucose and leukocyte count), the remaining blood was centrifuged (2000 x g, 10 minutes, 4ºC) and stored at -80ºC for future analysis.^40^

##### Clinical data collection

Data regarding patient demographics, medical history, medication use, intoxications and the American Society of Anaesthesiologists (ASA) score were collected from patient records. For the medical history of participants, the diagnosis and date were reported and the conditions were categorized by one of the two coordinating investigators of the ABC into the following categories: general, cardiovascular, respiratory, gastrointestinal, neurological, endocrinological, urological, locomotor, haematological, and psychiatric medical history.

In addition, surgical and perioperative anaesthetic data were collected, and data regarding biomaterial handling were registered. Preoperatively, neurocognitive function was tested with the Montreal Cognitive Assessment (MoCA) and a screening neurological examination was made if logistically possible. For the neurological examination a checklist consisting of 26 items (see Table S1) was followed to test for cranial nerve function, sensory and motor function, coordination, gait, and deep tendon reflexes. The entire examination was marked as abnormal if one or more items for that aspect were tested as abnormal.

#### Outcome and study procedures for the current study

##### Patient selection

At the time of this study, 486 patients had been included in the ABC. Data from all these patients, CSF from 405 and plasma from 478 were available for analysis in the current study.

From this cohort a control subpopulation was selected for the determination of preliminary reference intervals (central 95% of data).^30^^,^^31^ The controls were selected by excluding patients based on the following criteria: ASA Physical status III (patients with severe systemic disease with substantive functional limitations) and IV (patients with severe systemic disease that is a constant treat to life); a neurological, psychiatric or autoimmune disorder; severe cardiovascular of kidney disease; use of neuropsychiatric medication; excessive smoking (more than five cigarettes daily), alcohol abuse (more than two units per day) or recreational drug use; drinking or smoking habits unknown, and a current or past diagnosis of a malignant tumor. The classification of autoimmune diseases, which correspond to multiple medical history categories, was done according to the International Classification of Diseases (ICD) 10 list of autoimmune diseases.^41^

##### Additional laboratory analyses

CSF and plasma sCD27, plasma creatinine and C-reactive Protein (CRP) concentrations were measured by the Department of Clinical Chemistry at Uppsala University, Sweden.

For CSF, 300 μL of the third 2 mL fraction was used if available, otherwise CSF of the second 2mL fraction was used. sCD27 was analysed by a commercial sandwich ELISA kit (DY382, R&D Systems, Minneapolis, MN, USA) and all measurements were performed within the same batch. Details of the sCD27 analyses were described previously.^18^ In brief microtiter plates were coated with a monoclonal antibody specific for the peptide, and the wells blocked with bovine serum albumin. Samples were then pipetted into the wells. After washing a biotinylated anti-peptide antibody was added to the wells. Another incubation and washing cycle was performed and a streptavidine-HRP conjugate was added. Finally, after incubation and washing, a substrate solution was added. The development was stopped and the absorbance was measured in a SpectraMax 250 (Molecular Devices, Sunnyvale, CA, USA). The peptide concentrations were determined by comparing the optical density of the sample with the standard curve. The limit of detection (LOD) was 50 pg/mL and the highest standard point was 400,000 pg/mL. Eight calibrator points were added in duplicates to all plates. The samples were analyzed as singletons and the calibrator curve in the plate was used to calculate the CD27 concentration of the samples in the plate. The samples were analyzed during 3 consecutive days with 4 plates per day with the same calibrator and reagent batch to reduce assay variation. In this experiment the total CV was approximately 6% and the intraplate CV was approximately 4%.

Plasma creatinine was analysed with IDMS calibrated enzymatic creatinine reagents from Abbott Laboratories (Abbott Park, IL, USA) and analysed on a BS380 analyser (Mindray, Shenzhen, China). Plasma high-sensitivity CRP (hs-CRP) was analysed with Abbott Laboratories (Abbott Park, IL, USA) reagents on a BS380 analyser (Mindray, Shenzhen, China) and the LOD was 0.2 mg/L.

CSF/plasma quotient of albumin (Qalb ratio), an indicator of blood-CSF-barrier (BCB) function, and the CSF/plasma quotient of sCD27 (QsCD27 ratio) were calculated. The Qalb and QsCD27 ratios were calculated by dividing the concentration of albumin and sCD27 in CSF by the concentration of albumin and sCD27 in blood respectively, times one thousand. The ratio between QsCD27 and Qalb was calculated, to correct sCD27 values for blood-CSF-barrier function.

### Quantification and statistical analysis

Analyses were performed using SPSS statistics software (version 23.0; IBM Corp., Armonk, New York, Unites Stated of America). Data are expressed as mean ± standard deviation (SD) and as median [inter quartile range (IQR) or range] when appropriate. Discrete data are displayed by category frequencies and percentages. A p-value < .05 (for a two-tailed test) was considered statistically significant. If the sCD27 concentrations exceeded the upper limit of detection of 400,000 pg/mL or were below the lower limit of detection (LLD) of 50 pg/mL, the values were replaced with 401,000 pg/mL or 25 pg/mL (LOD/2), respectively, and nonparametric statistical tests were performed. If CRP concentrations were below the LLD of 0.2 mg/L, they were replaced with 0.1 mg/L. CSF sCD27 was log transformed. For comparisons between two groups, the Mann-Whitney U test was used. A Spearman rank correlation was performed when examining the relationship between two continuous variables.

The relationship between potential factors such as age, sex, Qalb, MoCA score and medical history category, and CSF sCD27 and plasma sCD27 concentrations was assessed in the total study group to determine the best subset of variables explaining CSF sCD27 levels or plasma sCD27 levels. A lasso regression model was built using R 4.0.3.,^42^ to accurately predict the combined relationship between sCD27 and multiple factors by simultaneously fitting the model and selecting variables to include through *shrinkage*.^43^ The optimal degree of shrinkage was been selected using leave-one-out cross-validation. For both CSF and plasma sCD27, twenty-six variables (e.g. age, BMI, CRP) were chosen to enter the model, and selection was based on the univariate analysis and logical reasoning. Finally, a Bayesian lasso^44^ was performed to compute the posterior probabilities for the coefficients of the lasso regression, as an alternative to p-values. The model was been fitted using the monomvn package,^45^ using Reversible Jump MCMC and the non-informative priors from Park & Casella.^44^

#### Sample size

A univariate and Bayesian lasso regression analysis was used to study the association between different factors and sCD27 concentrations. For this analysis we estimated that at least 261 patients were required to detect an effect size of 0.15 with a power of 95% and α = 0.05, using 30 variables.^46^
