# Supplementary material for: Cerebrospinal fluid and plasma concentrations of the inflammatory marker soluble CD27 in a large surgical population
Source: iScience. 2024 May 21;27(6):110036. doi: 10.1016/j.isci.2024.110036 (PMC11179565; doi:10.1016/j.isci.2024.110036)
Supplement: Document S1. Figures S1 and S2 and Tables S1–S4 [file mmc1.pdf]

iScience, Volume *27*

Supplemental information

Cerebrospinal fluid and plasma concentrations of the inflammatory marker soluble CD27 in a large surgical population

Celien Tigchelaar, Janet L. Cunningham, Annica J. Rasmusson, Måns Thulin, Joachim Burman, Ido P. Kema, Anders Larsson, and Anthony R. Absalom

Figure S1: Results from the Bayesian lasso regression model for predicting CSF sCD27(related to figure 1)

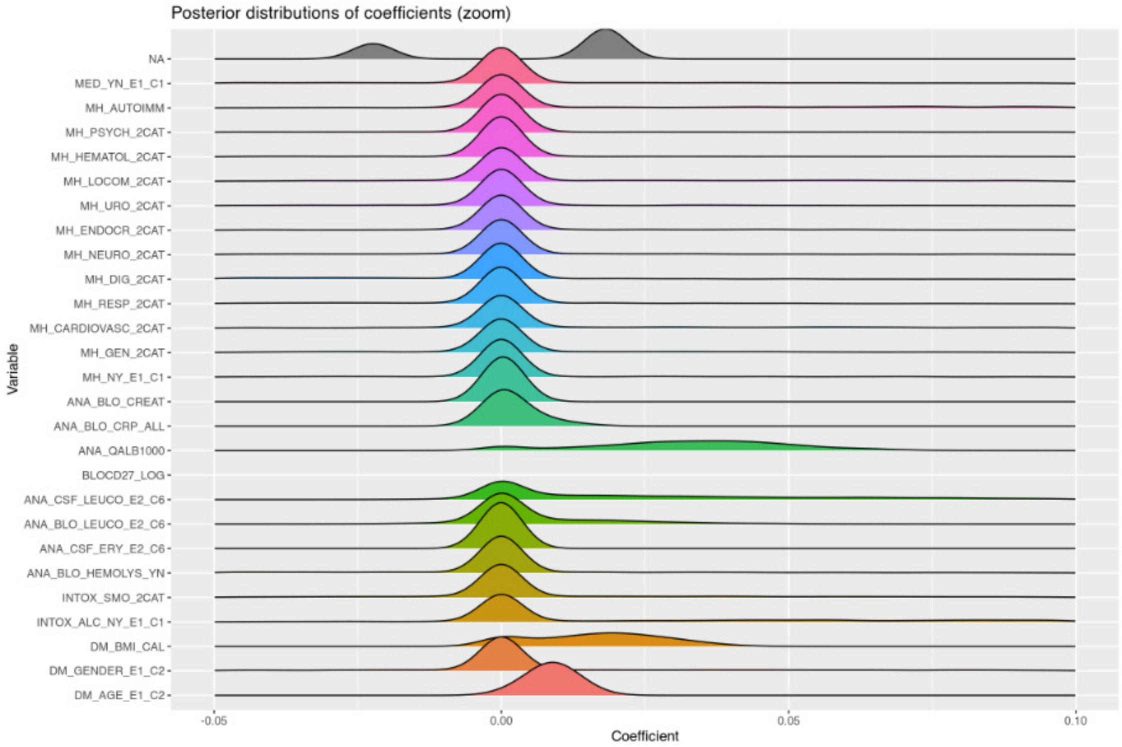

Figure S2: Results from the Bayesian lasso regression model for predicting plasma sCD27 (related to figure 1)

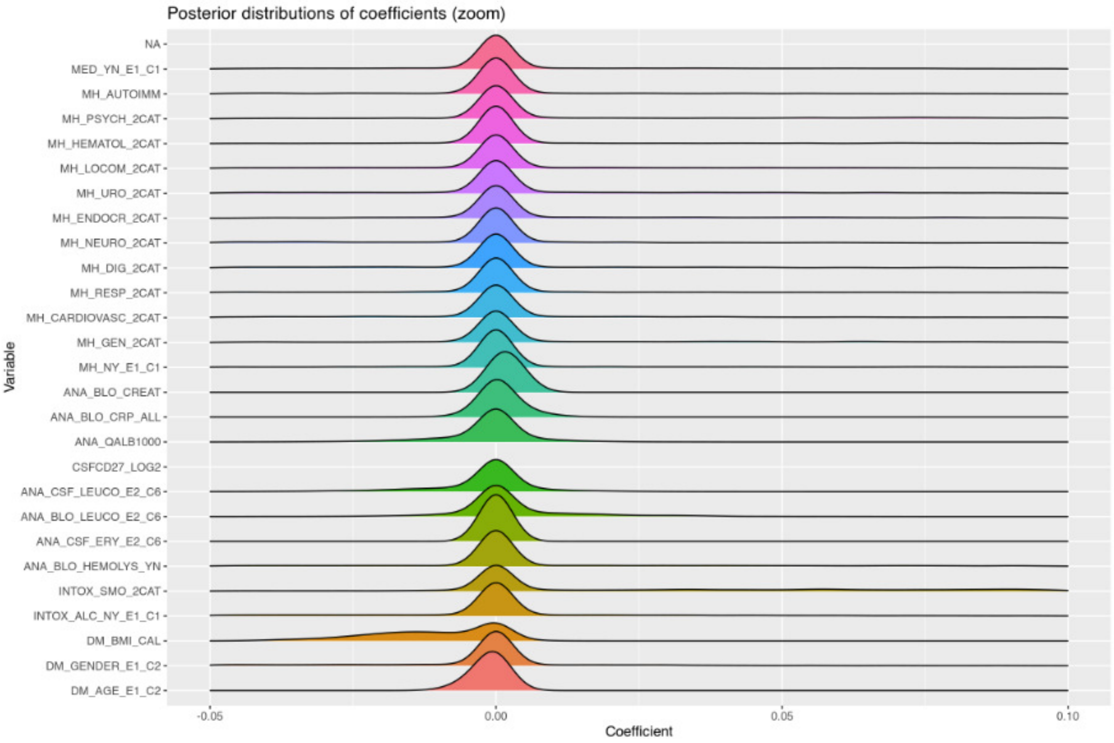

Table S1 Neurological examination (n=242) (for details of medical history see table 1 and 2)

	**Normal**		**Abnormal**		**Not tested**	
**Cranial nerve function**	**177**		**65**			
Visual field	230		12		0	
Eye inspection	231		11		0	
Pupil reflexes	238		4		0	
Eye movement	230		11		1	
Hearing	196		46		0	
Facial sensation	241		1		0	
Facial strength	242		0		0	
Facial expressions	240		2		0	
Palatal movement	242		0		0	
Shoulder/neck strength	239		3		0	
Tongue movement/strength	241		1		0	
**Motor function**	**168**		**74**			
Upper extremity strength	222		20		0	
Lower extremity strength	176		65		1	
Pronator drift	237		4		1	
**Sensory function**	**136**		**106**			
Sensation in extremities	143		99		0	
Romberg	222		13		7	
**Coordination**	**201**		**41**			
Finger tapping	227		14		1	
Rapid alternating movement	228		13		1	
Finger-nose test	233		9		0	
Heel-shin test	223		14		5	
**Gait**	165		67		10	
**Reflexes**	**79**					
**Reflexes**	**Absent**	**Reduced**	**Normal**	**Increased**	**Clonus**	**Not tested**
Biceps						
Right	21	10	208	1	0	2
Left	21	10	208	1	0	2
Triceps						
Right	68	23	147	1	0	3
Left	67	20	152	1	0	2
Knee						
Right	30	21	180	5	0	6
Left	29	15	187	8	0	3
Heel						
Right	66	23	145	2	0	6
Left	64	19	153	2	0	4
Plantar	**Indifferent**	**Abnormal**	**Normal**		**Not tested**	
Right	81	7	149		5	
Left	80	7	152		3	

Data are number of patients. For the main areas (cranial nerve function, motor function, sensory function, coordination, gait and reflexes), number of patients with normal and abnormal results for all items for that area are listed.

Table S2 The association between plasma sCD27 concentrations and physiological, social, cognitive pre-analytical and basic biochemical factors (see table 5 for associations with **CSF** sCD27)

**Study group (n=478)**		**Median[IQR] or frequency (%)**	**Plasma sCD27 median [IQR] (pg/mL)**	**Relationship with Plasma sCD27**	
				P value	r_s
Age (years)		57 [39 – 68]		0.014*	.112
Sex	Male	273 (57%)	4808 [3702 – 6857]	0.084	
	Female	205 (43%)	4419 [3341 – 7007]		
BMI (kg/m^2)		27 [24 – 31]		0.079	-.080
Alcohol use (n=437)	Yes	292 (67%)	4606 [3646 – 6756]	0.685	
	No	145 (33%)	4656 [3449 – 7364]		
Smoking (n=452)	Yes	72 (16%)	4475 [3619 – 6521]	0.688	
	No	380 (84%)	4626 [3531 – 6835]		
Plasma haemolysis	Yes	61 (13%)	4247 [3070 – 6379]	0.097	
	No	417 (87%)	4694 [3625 – 6871]		
Plasma leukocytes (n=471)		6.4 [5.4 – 7.8]		0.799	.012
CSF leukocytes (n=432)		1.0 [1.0 – 2.0]		0.063	-.090
Plasma albumin (n=475)		44 [42 – 46]		0.002**	-.141
Qalb x 10^{-3} (n=430)		5.4 [3.8 – 7.6]		0.053	.093
CSF CD27 (n=397)		161 [120 – 231]		< 0.001***	.284
Plasma CRP		1.26 [0.31 – 3.38]		0.037*	.096
Plasma creatinine		76 [65 – 89]		<0.001***	.238
MoCA (n=206)		26 [24 – 28]		0.377	-.057
Medical history	Yes	448 (94%)	4601 [3561 – 6875]	0.617	
	No	30 (6%)	4947 [3702 – 6442]		
General	Yes	263 (55%)	4652 [3645 – 7541]	0.193	
	No	215 (45%)	4574 [3374 – 6256]		
Cardiovascular	Yes	184 (38%)	4890 [3681 – 7528]	0.056	
	No	294 (62%)	4474 [3557 – 6331]		
Respiratory	Yes	100 (21%)	4496 [3417 – 6648]	0.451	
	No	378 (79%)	4652 [3598 – 7005]		
Gastrointestinal	Yes	111 (23%)	4873 [3734 – 7317]	0.336	
	No	367 (77%)	4589 [3524 – 6667]		
Neurological	Yes	90 (19%)	4736 [3768 – 6275]	0.809	
	No	388 (81%)	4616 [3485 – 6987]		
Endocrinological	Yes	106 (22%)	5357 [3716 – 7874]	0.043*	
	No	372 (78%)	4502 [3553 – 6756]		
Urological	Yes	107 (22%)	5158 [3963 – 7213]	0.022*	
	No	371 (78%)	4472 [3395 – 6811]		
Locomotor	Yes	284 (59%)	4514 [3553 – 6592]	0.409	
	No	194 (41%)	4810 [3630 – 7133]		
Hematological	Yes	18 (4%)	6874 [4944 –10413]	0.003**	
	No	460 (96%)	4570 [3531 – 6758]		
Psychiatric	Yes	42 (9%)	4499 [3642 – 7777]	0.372	
	No	436 (91%)	4626 [3568 – 6835]		
Autoimmune diseases	Yes	50 (10%)	5453 [3810 – 9514]	0.085	
	No	428 (90%)	4570 [3531 – 6648]		
Medication use	Yes	373 (78%)	4652 [3625 – 7015]	0.244	
	No	105 (22%)	4476 [3296 – 6628]		

Test is significant at 0.05 level (*),0..01 level (**) or 0.001 level (***) (two-tailed). r_s: Spearman correlation coefficient. BMI: Body Mass Index. Qalb: CSF albumin / plasma albumin. MoCA: Montreal Cognitive Assessment.

Table S3: Results of the Bayesian lasso regression model for predicting CSF sCD27 (related to figure 1)

Results from the Bayesian lasso regression model, including the posterior probabilities $P(\beta \neq 0|x)$ that the coefficients are non-zero:

	LASSO	BLASSO	Lower bound	Upper bound	P(beta≠0|x)
(Intercept)	1.664	2.155	1.042	3.258	NA
DM_AGE_E1_C2	0.010	0.009	0.002	0.014	0.975
DM_GENDER_E1_C2	0.032	0.000	-0.047	0.113	0.357
DM_BMI_CAL	0.021	0.017	0.000	0.034	0.846
INTOX_ALC_NY_E1_C1	0.144	0.045	0.000	0.237	0.632
INTOX_SMO_2CAT	0.000	0.000	-0.047	0.107	0.375
ANA_BLO_HEMOLYS_YN	-0.054	0.000	-0.190	0.055	0.380
ANA_CSF_ERY_E2_C6	0.000	0.000	0.000	0.000	0.619
ANA_BLO_LEUCO_E2_C6	0.010	0.000	-0.005	0.031	0.412
ANA_CSF_LEUCO_E2_C6	0.079	0.014	0.000	0.101	0.693
BLOCD27_LOG	1.056	1.028	0.793	1.271	1.000
ANA_QALB1000	0.038	0.034	0.000	0.061	0.928
ANA_BLO_CRP_ALL	0.005	0.000	-0.001	0.011	0.444
ANA_BLO_CREAT	0.001	0.000	-0.001	0.002	0.414
MH_NY_E1_C1	0.000	0.000	-0.120	0.238	0.377
MH_GEN_2CAT	0.000	0.000	-0.069	0.100	0.379
MH_CARDIOVASC_2CAT	0.027	0.000	-0.036	0.173	0.420
MH_RESP_2CAT	-0.048	0.000	-0.156	0.044	0.370
MH_DIG_2CAT	-0.204	-0.068	-0.330	0.000	0.641
MH_NEURO_2CAT	0.000	0.000	-0.086	0.122	0.366
MH_ENDOCR_2CAT	0.000	0.000	-0.056	0.172	0.377
MH_URO_2CAT	0.000	0.000	-0.086	0.096	0.329
MH_LOCOM_2CAT	0.001	0.000	-0.049	0.125	0.380
MH_HEMATOL_2CAT	-0.084	0.000	-0.295	0.109	0.368
MH_PSYCH_2CAT	0.000	0.000	-0.155	0.133	0.349
MH_AUTOIMM	0.291	0.089	0.000	0.442	0.643
MED_YN_E1_C1	0.000	0.000	-0.107	0.114	0.364

Table S4: Results from the Bayesian lasso regression model for predicting plasma sCD27 (related to figure 1)

Results from the Bayesian lasso regression model, including the posterior probabilities $P(\beta \neq 0|x)$ that the coefficients are non-zero:

	LASSO	BLASSO	Lower bound	Upper bound	P(beta≠0|x)
(Intercept)	10.078	10.106	9.367	10.852	NA
DM_AGE_E1_C2	0.000	0.000	-0.006	0.001	0.522
DM_GENDER_E1_C2	0.000	0.000	-0.155	0.034	0.445
DM_BMI_CAL	-0.013	-0.009	-0.031	0.000	0.701
INTOX_ALC_NY_E1_C1	0.000	0.000	-0.115	0.048	0.431
INTOX_SMO_2CAT	0.119	0.067	0.000	0.276	0.693
ANA_BLO_HEMOLYS_YN	0.000	0.000	-0.131	0.114	0.389
ANA_CSF_ERY_E2_C6	0.000	0.000	0.000	0.000	0.415
ANA_BLO_LEUCO_E2_C6	0.000	0.000	-0.009	0.025	0.431
ANA_CSF_LEUCO_E2_C6	0.000	0.000	-0.019	0.009	0.431
CSFCD27_LOG2	0.341	0.341	0.250	0.434	1.000
ANA_QALB1000	0.000	0.000	-0.016	0.009	0.419
ANA_BLO_CRP_ALL	0.000	0.000	-0.003	0.007	0.438
ANA_BLO_CREAT	0.002	0.002	0.000	0.005	0.743
MH_NY_E1_C1	0.000	0.000	-0.150	0.164	0.377
MH_GEN_2CAT	0.000	0.000	-0.043	0.169	0.453
MH_CARDIOVASC_2CAT	0.000	0.000	-0.101	0.083	0.420
MH_RESP_2CAT	0.000	0.000	-0.180	0.053	0.444
MH_DIG_2CAT	0.000	0.000	-0.105	0.099	0.403
MH_NEURO_2CAT	-0.081	0.000	-0.292	0.020	0.562
MH_ENDOCR_2CAT	0.000	0.000	-0.035	0.210	0.454
MH_URO_2CAT	0.000	0.000	-0.102	0.101	0.428
MH_LOCOM_2CAT	0.000	0.000	-0.074	0.083	0.381
MH_HEMATOL_2CAT	0.079	0.000	-0.059	0.478	0.501
MH_PSYCH_2CAT	0.202	0.082	-0.008	0.489	0.654
MH_AUTOIMM	0.000	0.000	-0.122	0.129	0.385
MED_YN_E1_C1	0.000	0.000	-0.091	0.140	0.421
